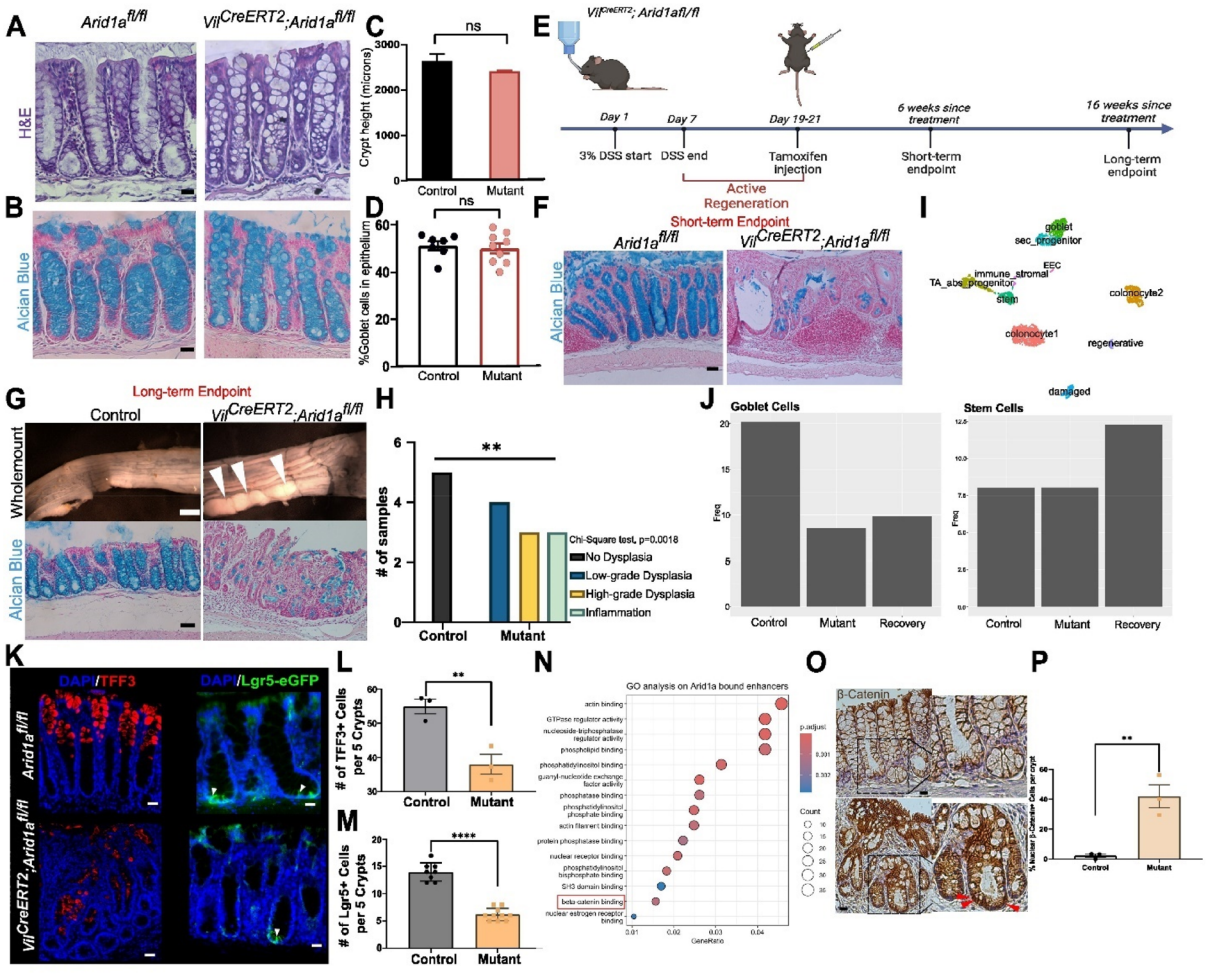# Poster Session I - Poster of Distinction I - A18 EXPLORING THE REGULATORY ROLE OF *ARID1A* IN ADULT COLONIC HOMEOSTASIS AND INJURY RESPONSE

**DOI:** 10.1093/jcag/gwaf042.018

**Published:** 2026-02-13

**Authors:** D Lei, A Loe, T Kim

**Affiliations:** Molecular Genetics, University of Toronto, Toronto, ON, Canada; Developmental and Stem Cell Biology, SickKids Research Institute, Toronto, ON, Canada; The Hospital for Sick Children, Toronto, ON, Canada

## Abstract

**Background:**

Recent advances in cancer genome analysis have illustrated the role of epigenetic regulators in cancer development. One regulator, ARID1A (AT-rich interactive domain containing protein 1A), a key component of the BAF (BRG1/BRM associated factor) chromatin remodeling complex, is frequently mutated in colorectal cancer (CRC), the third most prevalent cancer worldwide. Moreover, whole genome sequencing analysis has shown that *ARID1A* is highly altered in IBD patients along with other CRC-associated genes, such as *TP53* and *APC*. In fact, colonic injury resulting from conditions such as inflammatory bowel disease (IBD) is a significant risk factor for colorectal cancer, also known as colitis-associated cancer (CAC). Additionally, ARID1A is important for intestinal stem cell function during development, but its loss in epithelial and stromal tissue leads to colonic adenoma in adult mice. Therefore, the exact role of *ARID1A* in the adult colonic epithelia remains unclear.

**Aims:**

To comprehensively address the role of *ARID1A* in the adult colon, I aim to: 1. Determine the role of  *Arid1a*  during colonic homeostasis and injury. 2. Elucidate the regulatory mechanism by  *Arid1a*  during colitis.

**Methods:**

Tissue-specific *Arid1a* conditional KO mouse (*Villin-CreERT2; Arid1afl/fl*) was used for histological analysis after *Arid1a KO*. Additionally, after administering dextran sulfate sodium (DSS) to induce colitis-like injuries in the mouse colon, *Arid1a* was deleted during tissue regeneration and examined after short- and long-term.

**Results:**

While *Arid1a* deletion did not cause morphological differences in adult mice colon, *Arid1a*-deficient colons with DSS-induced colitis showed delayed recovery (Fig1A-F). Long-term *Arid1a-* colitis tissues showed visible tumor-like structures characterized by high-grade dysplasia, a hallmark event in CAC development (Fig1G-H). Single cell RNA- and ATAC-seq analyses revealed reduced stem- and goblet cells and downregulated motif enrichment of goblet cell lineage regulator, KLF4, in *Arid1a-* tissues, consistent with short-term goblet cell differentiation defect and stem cell number decrease *in vivo* (Fig1 I-M). Additionally, ARID1A and enhancer-specific ChIP-seq on recovered tissue revealed enrichment in β-catenin binding, suggesting *Arid1a* regulation of *Wnt* pathway activity during recovery. Indeed, long-term *Arid1a-* dysplastic tissues show higher nuclear β-catenin localization, suggesting overactivation of *Wnt* pathway in the absence of *Arid1a* (Fig1 N-P).

**Conclusions:**

1. *Arid1a* is important for colonic injury recovery, and its loss results in short-term recovery defects and long-term tumorigenesis. 2. *Arid1a* may interact with KLF4 to regulate goblet cell differentiation and regulate Wnt pathway activation during injury recovery.

**Funding Agencies:**

CIHR